# Measles outbreak in Northern Central African Republic 3 years after the last national immunization campaign

**DOI:** 10.1186/1471-2334-13-103

**Published:** 2013-02-26

**Authors:** Vianney Tricou, Marilou Pagonendji, Casimir Manengu, Jeff Mutombo, Rock Ouambita Mabo, Ionela Gouandjika-Vasilache

**Affiliations:** 1 Institut Pasteur de Bangui, Bangui, Central African Republic; 2 WHO Office, Bangui, Central African Republic; 3 Médecins Sans Frontières Spain, Bangui, Central African Republic; 4 Ministère de la Santé Publique, de la Population et de la Lutte contre le SIDA, Bangui, Central African Republic

**Keywords:** Measles, Outbreak, Central African Republic, Supplementary immunization activities

## Abstract

**Background:**

Despite huge efforts to promote widespread vaccination, measles remains an important cause of morbidity and mortality worldwide, especially in African children. In March 2011, an abnormally high number of cases were reported from the Ouham Prefecture, Central African Republic to the national measles case-based surveillance system. In response, reactive vaccination activities were implemented. The aims of this study were to investigate this outbreak and describe the response.

**Methods:**

Measles cases were defined according to WHO recommendations. In the first weeks of the outbreak, blood samples were collected and sent to the Institut Pasteur in Bangui for laboratory confirmation by detection of IgM antibodies against measles virus. In addition, a portion of viral RNA was amplified from 5 IgM positive patient samples and the amplicons were sequenced for phylogenetic analysis.

**Results:**

Between March and September 2011, 723 clinical cases originated from the Ouham Prefecture, including 2 deaths, were reported. Amongst 59 blood samples collected, 49 were positive for the detection of IgM. A high number of self-declared vaccinated subjects (31%) were found amongst the cases. Most of the cases were under 5 years. The causative virus was found to belong to genotype B3.1. In response, 2 sub-national supplementary immunization activities were quickly conducted and limited this outbreak to mainly 2 sub-prefectures.

**Conclusions:**

This outbreak was the largest epidemic of measles in CAR since 2002. Its occurrence, 3 years after the last national immunization campaign, highlights the necessity to pursue efforts and improve and extend immunization programs in order to reach measles elimination goal in Africa.

## Background

Measles is a highly contagious viral disease, which affects the respiratory system, mostly in children. Although most people recover within 2–3 weeks, serious complications and even death can occur. In developing countries, observed high case-fatality rates are due to a young age at infection, crowding, poor healthcare, malnutrition and underlying immune deficiency (e.g. AIDS) [1]. There is no specific treatment, but measles can be prevented by immunization. Huge efforts have been made to promote widespread vaccination, especially in Africa, where the WHO/UNICEF estimates of immunization coverage with a first dose of measles-containing vaccine (MCV1) provided through routine infant immunization scheduled at 9 months of age, and measured by one year of age increased from 56% to 76% between 2000 and 2010 [2]. During the same period, Supplementary Immunization Activities (SIA) resulted in vaccination of approximately half a billion children in Africa, and the number of measles cases reported decreased by 62%, from 520,102 in 2000 to 199,174 in 2010 [2,3]. Despite these efforts, measles remains an important cause of morbidity and mortality worldwide, especially in African children [4]. Another snag is the recent resurgence of measles in Europe despite substantial progress made towards measles elimination but where the insufficient vaccination coverage has allowed for silent accumulation of susceptible individuals [5].

The Central African Republic (CAR), one of the poorest countries of the world, is affected by political instability and internal conflicts for more than a decade. In northern CAR, close to 200,000 persons have been displaced between 2005 and 2008 and micro-displacement continues to this date due to constant insecurity.

In CAR, measles was considered “endemo-epidemic” until 2005. The total number of reported cases varied from 3207 in 2000 to 1233 in 2004, then 471 in 2005 [6]. Between 2006 and 2010, number of annually reported cases was low (less than 50 cases per year). Two campaigns of National Immunization Days (NID) were held, one catch-up campaign in 2005 targeting children aged 6 months to 14 years, and one follow-up campaign in 2008 targeting children between 9 and 59 months. The estimated vaccination coverage on these campaigns was 92% and 102%, respectively (unpublished Ministry of Health data). The WHO/UNICEF estimates of annual coverage with MCV1 in children under 1 year varied from 36% in 2000 to 62% in 2010 in CAR [7].

**Figure 1:**
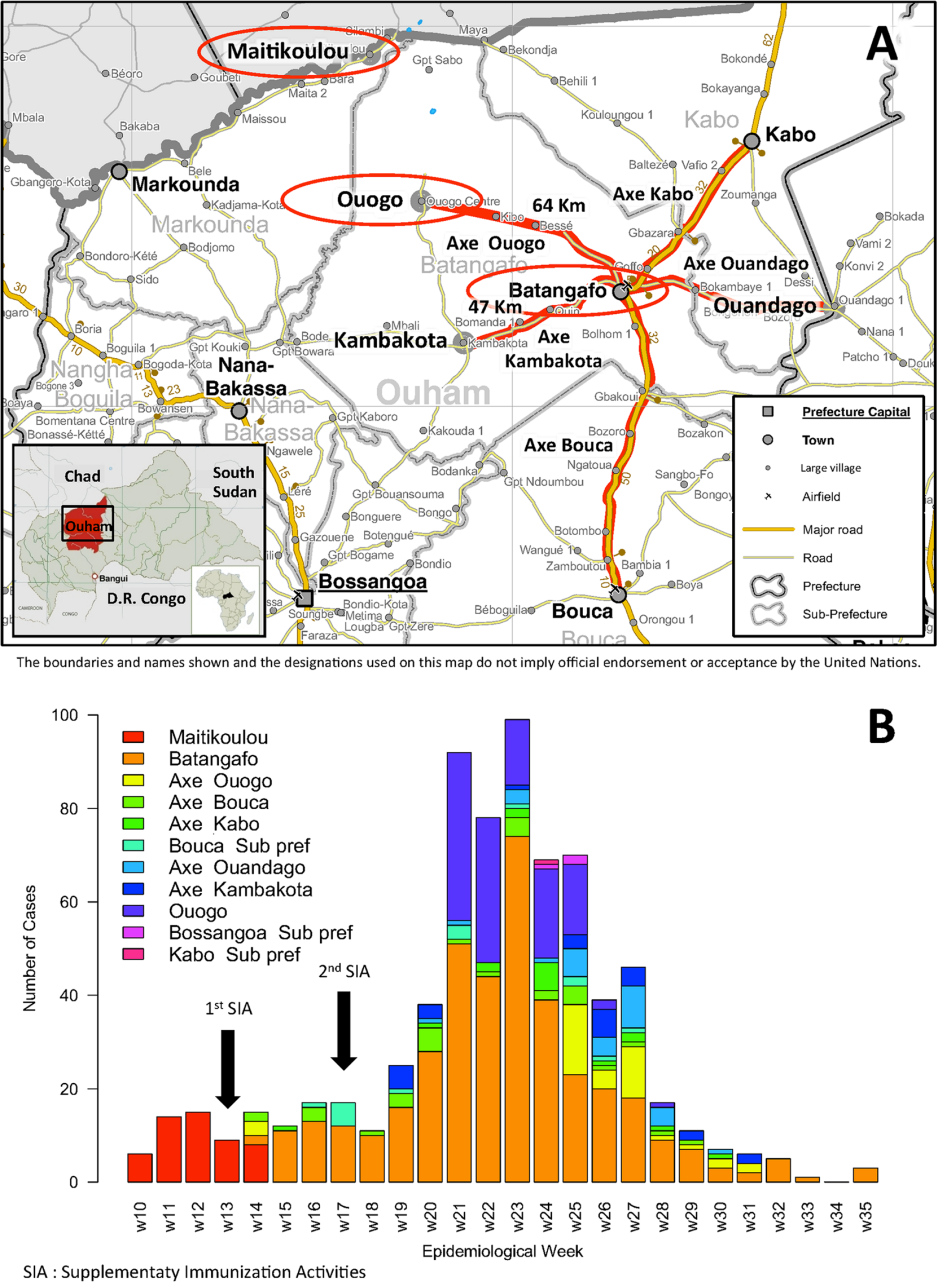
**Geographical location of the outbreak (A), and epidemic curve of measles suspected cases (B), Ouham Prefecture, Central African Republic, 2011. **The outbreak took place in the Ouham Prefecture in northern CAR. The main outbreak foci and links between them are highlighted in red. Suspected cases were reported between the 10th to the 35th epidemiological weeks of 2011. The 2 subnational SIA conducted from March 28 to April 1, 2011 (epi week 13) in Maitikoulou, Markounda Sub-prefecture (1st SIA) and from April 25 to April 29 (epi week 17), 2011 in Batangafo Sub-prefecture (2nd SIA) were represented by arrows. Blank map has been produced by UNDP on behalf of the Humanitarian and Development Partnership Team in Central African Republic (HDPT CAR) | Bangui, Central African Republic | 6 May 2008 | http://www.hdptcar.net. Map sources are GAUL, SIGCAF, HDPT CAR. Reprinted with the permission from the UNDP. The findings, interpretations and conclusions are strictly those of the authors and do not necessarily represent the views of UNDP or United Nations Member States.

In March 2011, an abnormally high number of clinical cases were reported in the Ouham Prefecture in northern CAR by the national measles case-based surveillance system (Figure 1). The aims of this study were to investigate this outbreak and describe the implemented response.

## Methods

### Ethics statement

This was a non-research national public health surveillance activity approved by the Ministry of Public Health, Population and the Fight Against AIDS of CAR. Approval by institutional review board or written informed consent was not required.

### Case definitions and classification

The case-based surveillance requires that every case presenting with fever and maculopapular rash (i.e. non-vesicular) and any of the following: cough, coryza (i.e. runny nose) or conjunctivitis (i.e. red eyes) is investigated and laboratory-tested. Measles surveillance is conducted through sentinel site reporting. In the Ouham Prefecture, there were 30 reporting sites (the same of the acute flaccid paralysis surveillance system). After laboratory-testing 5 to 10 cases closely related in time and space and finding 60% or more positive, an outbreak is declared and further laboratory-testing stops and related cases are considered as epidemiologically linked confirmed. We reviewed information about all the cases originated from the Ouham Prefecture in northern CAR reported to the national measles case-based surveillance system between March and September 2011. Cases were defined according to WHO recommendations and classified as suspected, clinical, laboratory confirmed, epidemiologically linked confirmed, clinically compatible or discarded [8].

### Laboratory diagnosis and virus genotyping

Blood samples from the first suspected cases were collected and sent by Médecins Sans Frontières (MSF) to the Institut Pasteur in Bangui. MSF run the hospitals of Maitikoulou and Batangafo, and was involved in measles case-based surveillance by detecting and investigating the sporadic suspected cases in the area. Laboratory confirmation was done by detection of IgM antibodies against measles virus (ELISA, Dade Behring, ref. OWLI15). Once the Ministry of Public Health, Population and the Fight Against AIDS of CAR and the WHO declared the outbreak (following the laboratory confirmation of a sufficient number of closely related cases), MSF did the field investigations and participated in the outbreak response.

In addition, throat, urine and saliva samples were collected for later virus isolation and genotyping. We amplified a 526-bp portion of the N gene from 5 samples (3 urine and 2 serum samples) from patients with IgM antibodies and compared the sequences with the WHO reference sequences that are recommended for genotyping [9,10]. Phylogenetic analysis was conducted in MEGA5 [11].

## Results

### Description of outbreak

In Markounda Sub-prefecture, Ouham Prefecture where the outbreak started, the index case was an unvaccinated 6-year-old boy from Maitikoulou village. He was admitted on March 10 (epi week 10) to the local health facility run by MSF. Laboratory confirmation was done by the Institut Pasteur in Bangui on March 21. A total of 53 suspected measles cases were reported in Maitikoulou and surroundings between March 3 and April 4 (epi week 9 to 14); 32 (including the index case) were laboratory confirmed, 9 were epidemiologically linked, and 4 were clinically compatible cases. No IgM antibody was found in 7 blood samples, and the corresponding cases were considered as discarded measles cases (2 were diagnosed as rubella). Another patient had no rash and was classified as discarded. A market is weekly held in Maitikoulou.

During April 2011, the outbreak extended to Bataganfo Sub-prefecture, Ouham Prefecture which is 157 Km from Maitikoulou village. Batangafo is an important place for the trading in the area. The index case was an unvaccinated 10-year-old girl, who was admitted on April 3 (epi week 14) to the local hospital run by MSF. The Institut Pasteur reported laboratory confirmation on April 7. A total of 652 clinical cases occurred mainly in the cities of Batangafo and Ouogo and also other communes or villages in Batangafo Sub-prefecture, of which 15 were laboratory confirmed. Three cases were negative for IgM antibodies against measles virus and considered as discarded cases. The other cases were all classified as compatible clinical cases because of difficulties to fully investigate the epidemiological link due to limited and overwhelmed medical capacities in a context of humanitarian emergency.

Cases also occurred in 3 other sub-prefectures in Ouham Prefecture during the same period: 15 cases in Bouca (1 laboratory confirmed and 14 clinically compatible), 3 compatible cases in Bossangoa and 1 in Kabo (laboratory confirmed).

In total, 49 laboratory confirmed, 9 epidemiologically linked confirmed and 654 clinically compatible cases were recorded between March and September 2011 in the Ouham Prefecture. Most of the cases were from 2 sub-prefectures: Markounda and Batangafo at the border with Chad (Figure 1A). Among the 49 laboratory confirmed cases, 7 have been vaccinated within 5 days prior to sample collection. Most of the children involved were under 5 years (412/723 i.e. 62%) with a sex ratio of 0.88 (male versus female) (Table 1). The epidemic peak was reached during the 23rd week, with 98 cases per week (Figure 1B).

**Table 1:** Case distribution by age and vaccine status, measles outbreak, Ouham Prefecture, Central African Republic, 2011

**Vaccination status**	**Age of cases**				**Total**
	**<1 year**	**1-4 years**	**5-14 years**	**≥15 years**	
Unknown	4 (1.7%)	15 (7.4%)	19 (12.1%)	4 (3.3%)	42 (5.8%)
Unvaccinated	176 (72.7%)	105 (51.5%)	86 (54.8%)	90 (75.0%)	457 (63.2%)
Vaccinated	62 (25.6 %)	84 (41.2%)	52 (33.1%)	26 (21.7%)	224 (31.0%)
Total	242	204	157	120	723

### Genotyping

**Figure 2:**
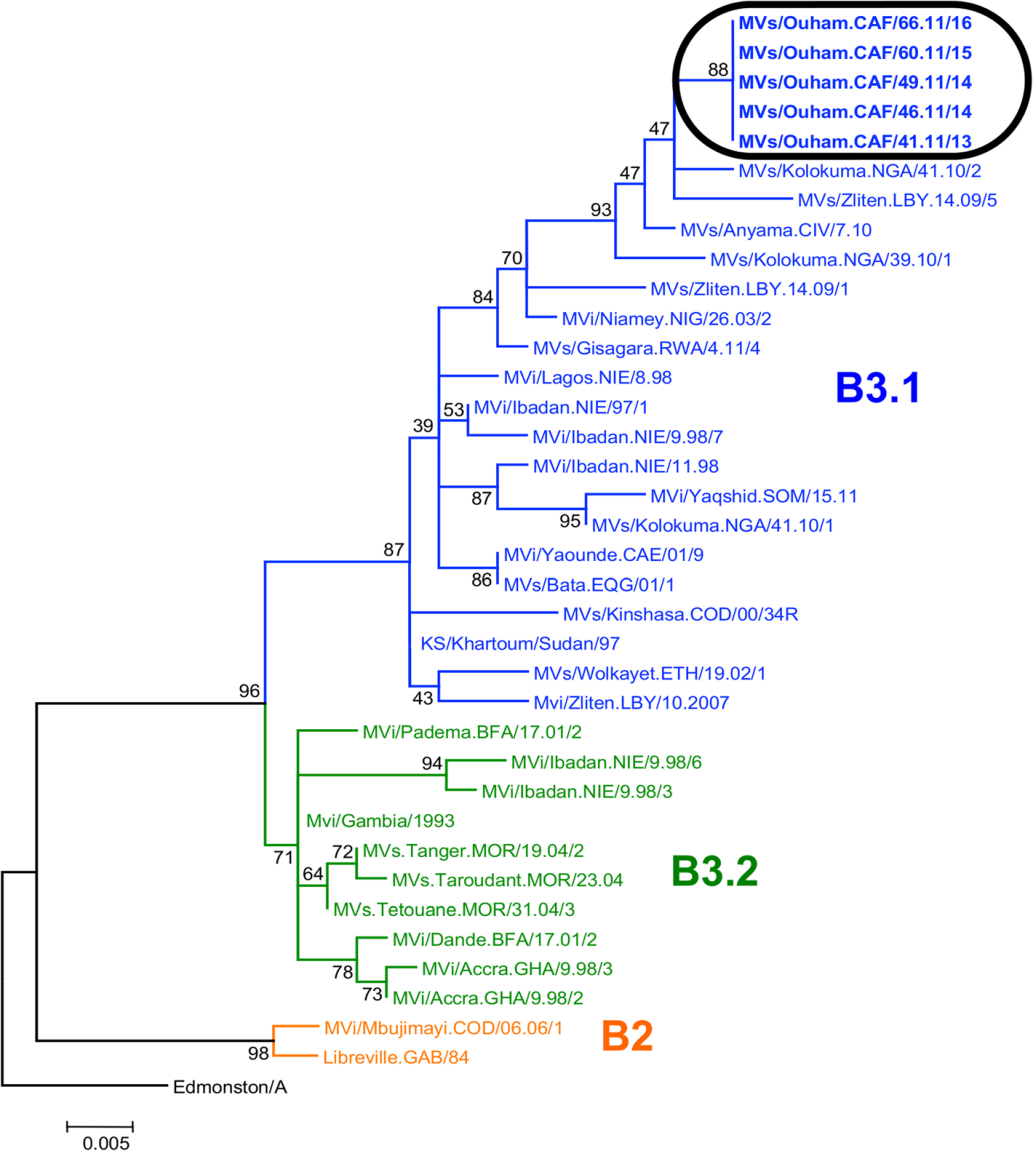
**Molecular phylogenetic analysis of virus isolates, Ouham Prefecture, Central African Republic, 2011. **The neighbor-joining method was used for reconstructing the phylogenetic tree of the N gene sequences of reference B2 and B3 measles virus strains and new sequences from the Central African Republic identified in this study (surrounded). The percentages of replicate trees in which the associated virus isolates clustered together in the bootstrap test (1000 replicates) are shown next to the branches.

The 5 sequences [GenBank:JQ669934, GenBank:JQ669935, GenBank:JQ669936, GenBank:JQ669937, GenBank:JQ669938] were 100% similar and fell within subgroup B3.1 (Figure 2).

### The response

Subnational SIA were conducted from March 28 to April 1 (epi week 13) in Maitikoulou village, Markounda Sub-prefecture and from April 25 to April 29 (epi week 17) in Batangafo Sub-prefecture targeting, respectively, 972 and 30 853 children from ages of 9 months to 15 years (Figure 1B). The estimated administrative coverage was respectively 116% in Maitikoulou, Markounda Sub-prefecture and 92% in Batangafo Sub-prefecture.

## Discussion

This outbreak was the largest epidemic of measles in CAR since 2002 [12]. The concerned area is regularly affected by insecurity and people face enormous difficulties in accessing healthcare. Only NGOs like MSF provide health services to the population in this area.

This epidemic likely occurred because of poor vaccination coverage against measles. Indeed, without high coverage through routine services and however SIA, the population of susceptible infants and children rapidly expand, especially if migrant, and the probability of a large measles outbreak increases in case of virus reintroduction [13]. At the same time, a measles outbreak, that began earlier in 2011, was on-going in neighboring southern Chad (Logone Occidental Region) [14,15]. The border between Chad and CAR is porous. Every day, people cross over this border and some may have brought measles virus along with them into CAR but without in-depth case investigations and clear data on the outbreak that occurred in Chad, it is difficult to conclude whether the cases in CAR were endemic or linked to an importation from Chad. Recent successful interruptions of measles transmission in CAR plead in favor of an importation. Only measles viruses that belong to subgroup B3.1 have been found circulating in CAR since 2002. Data on viral sequence are important to monitor the strains circulation, especially in absence of sufficient vaccination pressure, and to show disappearance of existing cluster(s) in case of an outbreak response.

The high number of vaccinated amongst suspected cases (224/723 i.e. 31%) can be explained in part by the inaccurate reporting of vaccination status and by difficulties with ensuring a reliable vaccine cold chain.

The response limited this outbreak mainly to 2 sub-prefectures but did not stopped the transmission. In October and November 2011, 10 other laboratory confirmed cases occurred in Kabo Sub-prefecture, Ouham Prefecture, underlining the importance of pursuing efforts and extend the SIA to a larger area around the center of the outbreak. To maintain gains in measles control, 95% routine coverage is required, with periodic NID (every 2–3 years) as recommended by WHO [16]. Surveillance standards must also be maintained to ensure rapid detection of measles cases, thus allowing a timely response and containment.

## Conclusions

This outbreak was the largest epidemic of measles in CAR since 2002. Its occurrence, 3 years after the last national immunization campaign, highlights the necessity to pursue efforts and improve and extend immunization programs in order to reach measles elimination goal in Africa.

## Abbreviations

MCV1: First dose of measles-containing vaccine; MCV2: second dose of measles-containing vaccine; SIA: Supplementary immunization activities; CAR: Central African Republic; NID: National immunization days; IgM: ELISA; MSF: Médecins Sans Frontières; NGOs: Non Governmental Organizations

## Competing interests

The authors declare that they have no competing interests.

## Authors’ contributions

VT participated in the analysis and the interpretation of the data and drafted the manuscript. MP participated in the study design, the experimental work, the analysis and the interpretation of the data. CM participated in the study design, the data collection and the analysis and interpretation of the data. JM and ROM participated in the data collection and revising of the manuscript. IGV conceived and designed the study and participated in the analysis and interpretation of the data and writing of the manuscript. All authors have read and approved the final manuscript.

## Pre-publication history

The pre-publication history for this paper can be accessed here:

http://www.biomedcentral.com/1471-2334/13/103/prepub
